# Genome‐Wide Association Study of Pericardial Fat Area in 28 161 UK Biobank Participants

**DOI:** 10.1161/JAHA.123.030661

**Published:** 2023-10-27

**Authors:** Ahmed Salih, Maddalena Ardissino, Aaron Z. Wagen, Andrew Bard, Liliana Szabo, Mina Ryten, Steffen E. Petersen, André Altmann, Zahra Raisi‐Estabragh

**Affiliations:** ^1^ William Harvey Research Institute, National Institute for Health and Care Research (NIHR) Barts Biomedical Research Centre Queen Mary University of London, Charterhouse Square London United Kingdom; ^2^ National Heart and Lung Institute, Imperial College London London United Kingdom; ^3^ Heart and Lung Research Institute, University of Cambridge Cambridge United Kingdom; ^4^ Genetics and Genomic Medicine, Great Ormond Street Institute of Child Health University College London London United Kingdom; ^5^ Department of Clinical and Movement Neurosciences Queen Square Institute of Neurology London United Kingdom; ^6^ Neurodegeneration Biology Laboratory The Francis Crick Institute London United Kingdom; ^7^ Barts Heart Centre, St Bartholomew’s Hospital, Barts Health National Health Service (NHS) Trust, West Smithfield London United Kingdom; ^8^ Semmelweis University, Heart and Vascular Center Budapest Hungary; ^9^ NIHR Great Ormond Street Hospital Biomedical Research Centre University College London London United Kingdom; ^10^ Health Data Research UK London United Kingdom; ^11^ Alan Turing Institute London United Kingdom; ^12^ Centre for Medical Image Computing, Department of Medical Physics and Biomedical Engineering University College London London United Kingdom

**Keywords:** cardiovascular disease, genome‐wide association study, pericardial adipose tissue, Genetics

## Abstract

**BACKGROUND:**

Pericardial adipose tissue (PAT) is the visceral adipose tissue compartment surrounding the heart. Experimental and observational research has suggested that greater PAT deposition might mediate cardiovascular disease, independent of general or subcutaneous adiposity. We characterize the genetic architecture of adiposity‐adjusted PAT and identify causal associations between PAT and adverse cardiac magnetic resonance imaging measures of cardiac structure and function in 28 161 UK Biobank participants.

**METHODS AND RESULTS:**

The PAT phenotype was extracted from cardiac magnetic resonance images using an automated image analysis tool previously developed and validated in this cohort. A genome‐wide association study was performed with PAT area set as the phenotype, adjusting for age, sex, and other measures of obesity. Functional mapping and Bayesian colocalization were used to understand the biologic role of identified variants. Mendelian randomization analysis was used to examine potential causal links between genetically determined PAT and cardiac magnetic resonance–derived measures of left ventricular structure and function. We discovered 12 genome‐wide significant variants, with 2 independent sentinel variants (rs6428792, *P*=4.20×10^−9^ and rs11992444, *P*=1.30×10^−12^) at 2 distinct genomic loci, that were mapped to 3 potentially causal genes: T‐box transcription factor 15 (*TBX15*), tryptophanyl tRNA synthetase 2, mitochondrial (*WARS2*) and early B‐cell factor‐2 (*EBF2*) through functional annotation. Bayesian colocalization additionally suggested a role of RP4‐712E4.1. Genetically predicted differences in adiposity‐adjusted PAT were causally associated with adverse left ventricular remodeling.

**CONCLUSIONS:**

This study provides insights into the genetic architecture determining differential PAT deposition, identifies causal links with left structural and functional parameters, and provides novel data about the pathophysiological importance of adiposity distribution.

**Nonstandard Abbreviations and Acronyms**
CADD: combined annotation dependent depletion
MR: Mendelian randomization
PAT: pericardial adipose tissue
PC: principal component
PPH: posterior probability of hypothesis
WHR: waist:hip ratio

**CLINICAL PERSPECTIVE**

**What Is New?**

- This study identifies multiple distinct genetic loci associated with pericardial fat area after accounting for multiple measures of whole‐body adiposity.
- Mendelian randomization analyses identified an association of likely causal relevance of genetically predicted pericardial fat with adverse cardiac structural and functional parameters.

**What Are the Clinical Implications?**

- In addition to being determined by whole‐body adiposity, this study suggests that the proportional deposition of pericardial adipose tissue is, to an extent, genetically determined.
- Greater genetically predicted pericardial adipose tissue is linked with markers of adverse left cardiac structure and function, suggesting a role in determining adverse left ventricular remodeling.

Pericardial adipose tissue (PAT) is the visceral adipose tissue compartment surrounding the heart. Experimental research has suggested that a proportionally greater deposition of PAT might mediate the risk of cardiovascular disease in addition to that conferred by general adiposity through paracrine proinflammatory effects of the fat tissue on adjacent myocardium and coronary arteries.^1^, ^2^, ^3^, ^4^ In line with this, observational studies have reported associations between PAT and the risk of coronary artery disease,^5^ heart failure,^6^ atrial fibrillation^7^, ^8^ and adverse imaging markers of cardiac structure and function^9^, ^10^ even after an adjustment for multiple measures of general adiposity and its visceral and subcutaneous tissue distribution.

Body fat distribution is a highly heritable trait, with twin‐based estimates for body mass index (BMI)–adjusted waist:hip ratio (WHR) estimated between 30% and 60%,^11^ and single nucleotide polymorphisms (SNP)–based heritability in the region of 20% to 50%.^12^ So far, BMI‐adjusted WHR^13^ has been the main focus of large‐scale studies exploring the genetic determinants of fat distribution. Consequently, the genetic architecture and disease consequences of this trait have been thoroughly explored.^14^, ^15^, ^16^, ^17^ On the other hand, current understanding of the genetic determinants of fat deposition specifically in the pericardial tissue, independent of general adiposity and its distribution, remains limited.

At present, only 2 genome‐wide association studies (GWAS) have evaluated the genetic determinants of PAT in relation to whole‐body adiposity.^18^, ^19^ The largest of these, carried out in 2017 by Chu et al, included 18 332 participants and discovered 3 genetic variants in distinct loci associated with PAT after height and weight adjustment: rs6587515 in *ENSA*, rs1650505 in *EBF1*, and rs10198628 in *TRIB2*.^19^ Genetic discovery in this field has been limited by the lack of large‐scale data. We recently developed a fully automated, quality‐controlled tool for PAT quantification from cardiac magnetic resonance (CMR) images,^20^ enabling extraction of PAT measurements in 42 598 participants in the UK Biobank, a large‐scale cohort study collecting clinical, genetic, imaging, and laboratory data from participants throughout the United Kingdom.

In this study, we employed UK Biobank data to investigate the genetic variants predisposed to the deposition of PAT independent of other measures of total adiposity and its distribution. We additionally leverage these variants to assess the causal role of PAT on left ventricular (LV) structure and function.

## Methods

### Data Access and Availability

This study was conducted using the UK Biobank under application 2964. The work is covered by ethical approval from the National Health Service National Research Ethics Service on June 17, 2011 (reference 11/NW/0382) and extended on June 18,2021 (reference 21/NW/0157). Written, informed consent was obtained from all participants.

The data produced from this study, including summary statistics, methods, and materials, will be returned to the UK Biobank. These will become available to all bona fide researchers for the purpose of health‐related research under approved applications, without preferential or exclusive access. Further details about application and access procedures are available at the UK Biobank website (http://www.ukbiobank.ac.uk/register‐apply/).

### Study Population

The UK Biobank is a population‐based cohort study based in the United Kingdom. Between 2006 and 2010, >500 000 participants aged 40 to 69 years were recruited and underwent a baseline assessment and regular integration of health outcomes through healthcare record linkage. The detailed study protocol is publicly available.^21^ The UK Biobank Imaging Study is an ongoing subset of the UK Biobank aiming to perform multiorgan magnetic resonance imaging of the heart, brain, and abdomen in a randomly selected 20% (n=100 000) subset of UK Biobank participants.

### Pericardial Fat Quantification

CMR scans were performed using 1.5 Tesla scanners (MAGNETOM Aera, Syngo Platform VD13A, Siemens Healthcare, Erlangen, Germany) in specific imaging units. Scanning was performed according to predefined protocols.^22^ PAT area was extracted from CMR 4‐chamber cine images in end diastole using an automated tool that has been developed and validated in the UK Biobank and an external cohort.^20^ This involves a neural network trained to perform fully automated PAT segmentation through a multiresidual U‐net architecture. It includes an in‐built quality‐control feature, which uses Dice scores as a measure of segmentation quality. In this analysis, we limited to scans with good segmentation quality (Dice score > 0.7). In the study population, PAT areas had a right‐skewed distribution and were therefore log‐transformed for linear modeling.

### Measures of Adiposity

A key aim of the study was to determine whether the relationship between PAT and cardiovascular phenotypes was distinct from other obesity measures. We considered anthropometric measures of obesity, impedance fat measures, and abdominal magnetic resonance imaging–derived measures of visceral and subcutaneous adiposity. BMI and WHR were calculated from UK Biobank body size measures. Bioelectrical impedance measures of obesity were derived using the Tanita BC418MA body composition analyzer as per UK Biobank protocols.^23^ We included whole‐body fat mass and trunk fat mass impedance measures. From abdominal magnetic resonance imaging (available for 15 518 participants), we selected abdominal subcutaneous, visceral adipose tissue, and total adipose tissue volume measures, which are only available for a subset of participants.^24^

### Genetic Data and Quality Control

Genotyping was performed in all consenting individuals. Genotypes were directly called using the 2 closely related arrays UK Biobank Axiom (Affymetrix, Santa Clara, California) and UK Applied Biosystems UK BiLEVE Axiom Array (BiLEVE) Axiom. Imputation was carried out using the Haplotype Reference Consortium and UK10K+1000Genomes (phase 3) reference panels.

### Genome‐Wide Association Study

For genome‐wide association analysis, participants were excluded if their genetic samples failed bioinformatic quality control (missing rate on autosomes of >0.2 or mismatch between reported and genetically inferred sex), or if they were related (based on a kinship matrix with threshold K>0.175) by excluding 1 of the pair. The cohort was restricted to individuals of European ancestry. After exclusion criteria were applied, 28 161 participants were included. Among the available imputed and genotyped variants, we restricted the analysis to autosomal variants with a minor allele frequency>0.01 and imputation quality score (information score) >0.3. This resulted in ≈9 283 970 million variants. Genome‐wide association analysis was performed using PLINK^25^ and BOLT‐linear mixed model (BOLT‐LMM).^26^

In the main model, we assessed the association between variants and PAT after adjusting for sex, age, age^2^, age*sex, 10 genetic principal components (PCs), assessment center, genotype array, BMI, WHR, whole‐body fat mass, trunk fat mass, and body fat percentage. In this analysis, PC analysis was applied to BMI, WHR, whole‐body fat mass, trunk fat mass, and body fat percentage to explain at least 90% of the variance, which resulted in 2 PCs that explained 99% of the variance in the included phenotypes. These 2 PCs were included when GWAS was run instead of the BMI, WHR, whole‐body fat mass, trunk fat mass, and body fat percentage. For this model, the population was randomly split into set of 18 774 participants for discovery and a replication set of 9 387 participants for replication. This is the primary analysis of the study.

For discovery analysis, the threshold for statistical significance was considered *P*<5×10^−8^ to account for multiple tests. Replication analyses were carried out for all genome‐wide significant variant associations in the primary model. For replication analysis, the statistical significance threshold was calculated using Bonferroni correction based on the number of variants tested for validation (*P*<0.05/n; where n=number of lead variants to validate).

To increase the power for detection of significant signals using the whole sample, we additionally performed a meta‐analysis GWAS by combining the GWAS summary statistics of the discovery and replication analyses. This analysis was conducted using the Metal tool.^27^

We have also carried out a more relaxed GWAS without adjustment for different fat measures. The analysis was adjusted for sex, age, age^2^, age*sex, 10 genetic PCs, assessment center, and genotype array.

### Functional Annotation

Functional mapping was carried out using functional mapping and annotation (FUMA) of GWAS version 1.5.0.^28^ Independent significant SNPs were defined as those associated with PAT in the primary discovery analysis model with *P*<5×10^−8^ that were correlated with *r* ^2^<0.6. Additional candidate SNPs were identified by extracting SNPs in linkage disequilibrium with these at *r* ^2^>0.6 using the 1000Genomes phase 3 European reference panel.^29^ Finally, lead SNPs were identified among the candidates as the uncorrelated (*r* ^2^<0.1) SNPs with prioritization of those with lowest *P* value for the association with PAT. For lead SNPs and any SNPs in linkage disequilibrium with these at *r* ^2^>0.8, all reported phenotypic associations were listed using the GWAS Catalog.^30^

The functional consequences of the candidate SNPs on genes were determined using ANNOtate VARiation (ANNOVAR).^31^ Deleteriousness score was described using combined annotation dependent depletion (CADD) scores (with scores>12.37 considered likely deleterious),^32^ and SNPs were annotated for regulatory functions using regulatory elements database (RegulomeDB) score,^33^ for 15‐core chromatin state using chromatin hidden Markov model (ChromHMM),^34^, ^35^ tissue‐specific expression quantitative trait loci (eQTLs),^36^ and for 3‐dimensional chromatin interactions using high‐throughput adaptation of chromosome conformation capture (Hi‐C) data.^37^

Gene mapping was performed using positional, eQTL, and chromatin interactions mapping. First, genomic risk loci near independent significant SNPs were outlined using a maximum distance of 10 kB. Within each risk locus, the SNP with the lowest *P* value was defined as the lead SNP for the locus. Probability of loss of function intolerance was annotated using probability of being loss‐of‐function intolerant (pLI) scores for coding genes,^38^ and with noncoding residual variation intolerance scores for noncoding genes.^39^ Multi‐marker Analysis of GenoMic Annotation (MAGMA) gene‐based analysis was performed to assess the association between protein coding genes and PAT.^40^ Because the input SNPs were mapped to 19 086 protein coding genes, genome‐wide significance for this analysis was Bonferroni corrected at *P* value=0.05/19086=2.620×10^−6^. Tissue‐specific eQTL mapping was then performed using data from single‐cell RNA sequencing^41^ in immune cells, and GTEx (Genotype‐Tissue Expression) Project version 8^36^ tissue‐specific eQTL data for arterial, adipose, and cardiac tissues. Finally, chromatin mapping was performed using tissue‐specific chromatin interaction (Hi‐C) data for the aorta, left ventricle, and right ventricle.^37^, ^42^, ^43^, ^44^

To understand putative biological mechanisms behind mapped genes, gene‐to‐function mapping was performed within FUMA and GWASAtlas. GTEx version 8^36^ data were used to visualize normalized tissue‐specific expression patterns for each gene. Differentially expressed gene set analyses were performed to test for differential expression of mapped genes across tissue types. Phenome‐wide associations were identified for all potentially causal genes using GWASAtlas.^45^ Finally, the IMPC (International Mouse Phenotyping Consortium) database was searched for information about previous mouse models for potentially causal genes.^46^

### Colocalization Analysis

To evaluate the probability that GWAS loci and eQTLs share a single causal variant, a colocalization analysis was performed using coloc (version 5.1.0.1) and colochelpR (version 0.99.1).^47^, ^48^ Cis‐eQTLs were derived from GTEx version 8.^36^, ^49^ GWAS loci within 1 Mb of the 11 significant GWAS SNPs were explored. Loci identified through chromatin mapping were not included as these were expected to have trans‐associations. Associations were explored in 7 GTEx tissues: aortic artery (N=387), coronary artery (N=213), tibial artery (N=584), subcutaneous adipose (N=581), visceral adipose (N=469), the cardiac atrial appendage (N=372), and the cardiac LV (N=386). The prior probability that any random SNP in the region is associated with the GWAS (p_1_) or eQTL (p_2_) was set to the default 10^−4^, whereas the prior probability that any random SNP in the region is associated with both traits (p_12_) was set to 10^−5^. A posterior probability of hypothesis (PPH) 4 measures the probability that a locus is colocalized as the result of a single causal variant, as opposed to 2 distinct causal variants (PPH3). A PPH4≥0.8 was considered significant. All colocalizations were subjected to sensitivity analyses using coloc's sensitivity function, which plots prior and posterior probabilities of each coloc hypothesis as a function of the p12 prior. This permits exploration of the robustness of results to changes in the p12 prior. Code for coloc analyses is openly available at https://github.com/aaronwagen/Pericardial_fat_gwas_coloc/.

### Heritability and Genetic Associations

We used CTG‐VL 0.5 beta (https://vl‐dev.genoma.io/updates) to estimate trait heritability and calculate genetic correlation between PAT and multiple disease phenotypes. These included adiposity traits (trunk fat mass as percentage, whole‐body fat mass), cardiovascular risk factors (hypertension, diabetes, obesity), and cardiovascular outcomes (coronary heart disease, coronary event, heart failure, stroke, atrial fibrillation and flutter, and cardiac death).

Mendelian randomization (MR) was performed to assess the causal relevance of PAT on multiple CMR markers of LV structure and function, motivated by the previously established observational evidence suggesting potential causal mechanisms.^9^ Genome‐wide significant (*P*<5×10^−8^), uncorrelated (*r* ^2^<0.001) variants for PAT were selected as instrumental variants. Instrument strength was quantified using *F* statistics. Gene‐outcome association data were extracted from summary statistics on GWAS of 45 504 UK Biobank participants by Pirruccello et al^50^ for indexed LV end‐diastolic volume, LV end‐systolic volume, LV stroke volume, and LV ejection fraction. Additional gene‐outcome association data were extracted from the GWAS of 16 923 participants for LV mass (LVM) and mass to end‐diastolic volume ratio (LV mass:LVEDV) by Aung et al.^51^ Inverse‐variance weighted MR with fixed effects was used for primary analysis. Single‐SNP analysis was performed using the Wald ratio method. Importantly, the data source for both gene‐exposure and gene‐outcome association estimates in this case is the UK Biobank cohort. Although the MR methods used are considered “2‐sample” methods, they have been demonstrated to be robust for individual‐level analysis when applied in the setting of large‐scale biobanks.^52^ All MR analyses were performed using the MendelianRandomization package (version 0.7.0)^53^ in RStudio (R version 4.1.2).^54^

## Results

### Genome‐Wide Association Study

#### Genetic Variants Associated With Pericardial Fat Independent of BMI and Other Fat Distribution Measures

We used previously validated, automated, and quality‐controlled tool to extract measures of PAT area in 28 161 UKB participants who were randomly split into a discovery set of 18 774 participants and a testing set of 9 387 participants.

In the genome‐wide association analysis in the discovery set, and after adjusting for sex, age, age^2^, age*sex, 10 genetic PCs, assessment center, genotype array, and 2 PCs reflecting BMI, WHR, whole‐body fat mass, trunk fat mass, and body fat percentage, a total of 11 genome‐wide significant variants were identified (rs11992444, rs6428792, rs10923752, rs10923748, rs6428794, rs12036872, rs4304634, rs764891110, rs4659150, rs4659146, rs2885227) as reported in Figure S1, Table S1, and Table 1. The QQ plot for the results is presented in Figure S2. Genomic inflation factor (lambda, λ) was 1.026, and λ1000 was 1.001.

**Table 1:** Genome‐Wide Significant Variants: Genome‐Wide Analysis Identified 11 Sentinel Variants That Were Genome‐Wide Significant (*P*<5×10^−8^)

SNP	Chromosome	Position	Allele 1	Allele 0	Allele 1 frequency	Missing rate	β	Standard error	*P* value
rs11992444	8	25 464 690	G	T	0.490	0.003	−0.012	0.002	1.30E‐12
rs6428792	1	119 656 867	G	A	0.380	0.006	−0.010	0.002	4.20E‐09
rs10923752	1	119 658 925	G	A	0.341	0.007	0.010	0.002	1.40E‐08
rs10923748	1	119 647 946	G	C	0.341	0.007	0.010	0.002	1.60E‐08
rs6428794	1	119 657 743	A	T	0.341	0.007	0.010	0.002	1.60E‐08
rs12036872	1	119 660 505	C	G	0.341	0.007	0.010	0.002	1.60E‐08
rs4304634	1	119 650 931	T	A	0.340	0.009	0.010	0.002	1.80E‐08
rs764891110	1	119 651 167	T	TTATGA	0.341	0.010	0.010	0.002	1.80E‐08
rs4659150	1	119 660 819	T	G	0.340	0.008	0.010	0.002	1.90E‐08
rs4659146	1	119 645 535	T	C	0.342	0.009	0.010	0.002	2.10E‐08
rs2885227	1	119 650 928	C	A	0.340	0.009	0.010	0.002	2.00E‐08

The table displays β coefficients with standard errors, and *P* value estimates. Allele 1 is the effect allele. SNP indicates single nucleotide polymorphism.

Among the discovered variants, 1 single variant was located on chromosome 8, rs11992444 (*P*=1.30×10^−12^), and 10 variants were located on chromosome 1, among which the variant with lowest the *P* value was rs6428792 (*P*=4.20×10^−9^). The association of all 11 genome‐wide significant variants with PAT was replicated in the replication set at the Bonferroni‐corrected *P* value threshold (*P*<0.0045), as reported in Table 2.

**Table 2:** Replication of Association Between Genome‐Wide Significant Variants and Adjusted Pericardial Fat Area in the Testing Set

SNP	Chromosome	Position	Allele 1	Allele 0	Allele 1 frequency	Missing rate	β	Standard error	*P* value
rs11992444	8	25 464 690	G	T	0.489	0.002	−0.015	0.002	5.00E‐11
rs6428792	1	119 656 867	G	A	0.380	0.007	−0.008	0.002	0.00078
rs10923752	1	119 658 925	G	A	0.339	0.008	0.007	0.002	0.0028
rs10923748	1	119 647 946	G	C	0.339	0.008	0.007	0.002	0.0026
rs6428794	1	119 657 743	A	T	0.339	0.008	0.007	0.002	0.0027
rs12036872	1	119 660 505	C	G	0.339	0.008	0.007	0.002	0.0027
rs4304634	1	119 650 931	T	A	0.338	0.009	0.007	0.002	0.0026
rs764891110	1	119 651 167	T	TTATGA	0.339	0.011	0.007	0.002	0.0025
rs4659150	1	119 660 819	T	G	0.338	0.008	0.007	0.002	0.0026
rs4659146	1	119 645 535	T	C	0.339	0.010	0.007	0.002	0.0021
rs2885227	1	119 650 928	C	A	0.338	0.009	0.007	0.002	0.0025

All variants passed replication at Bonferroni‐adjusted statistical significance threshold (*P*<4.5×10^−3^). SNP indicates single nucleotide polymorphism.

### Functional Annotation

**Figure 1:**
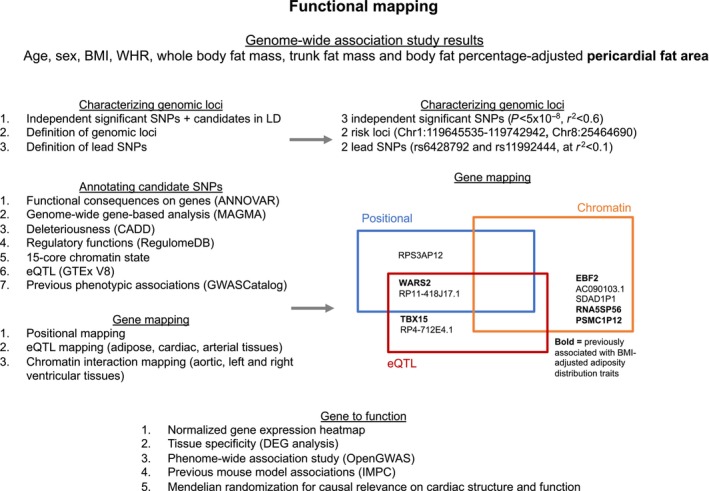
Methods and key results of functional annotation of genome‐wide significant variants and exploration of functional consequences of prioritized variants and genes. ANNOVAR indicates ANNOtate VARiation; BMI, body mass index; CADD, combined annotation dependent depletion; Chr, chromosome; DEG, differentially expressed gene; EBF2, early B‐cell factor‐2; eQTL, expression quantitative trait loci; GTEx, Genotype‐Tissue Expression; GWASCatalog, GWAS Catalog; IMPC, International Mouse Phenotyping Consortium; LD, linkage disequilibrium; MAGMA, Multi‐marker Analysis of GenoMic Annotation; OpenGWAS, IEU OpenGWAS project; PSMC1P12, proteasome 26S subunit, ATPase 1 pseudogene 12; RegulomeDB, regulatory elements database; RNA5SP56, RNA, 5S ribosomal pseudogene 56; SDAD1P1, SDA1 domain containing 1 pseudogene 1; SNP, single nucleotide polymorphism; TBX15, T‐box transcription factor 15; WARS2, tryptophanyl tRNA synthetase 2, mitochondrial; and WHR, waist:hip ratio.

Functional annotation through positional, eQTL, and chromatin interaction mapping identified a total of 10 potentially causal genes. A visual representation of the annotation process and key results are provided in Figure 1.

#### Positional Mapping

In addition to the 11 GWAS‐tagged variants, 1 additional closely correlated variant (rs72707349) was extracted using the 1000 Genomes reference panel. Among the 12 candidate SNPs, 2 lead variants were identified (*r* ^2^<0.1)—rs6428792 and rs11992444—in 2 separate genetic loci (Tables S1 through S3). All previously reported phenotypic associations for these 2 SNPs and SNPs in close linkage disequilibrium with these (*r* ^2^>0.8) are reported in Table S4, and these included multiple BMI‐adjusted adiposity traits, body shape indexes, and lipid traits.

Among the 12 candidate variants, the 11 variants on chromosome 1 were intronic (of which 1 in noncoding RNA), and the variant on chromosome 8 was intergenic (Table S5). RegulomeDB score for both variants was 7, indicating a lack of evidence about potential regulatory functions. The minimum 15‐core chromatin state was 5 for rs6428792, indicating weak transcription function, and 7 for rs11992444, indicating enhancer chromatin state. Positional mapping prioritized 3 genes: *WARS2* (protein coding), *RPS3AP12* (pseudogenic) and *RP11‐418J17.1* (antisense), all mapped to the chromosome 1 locus (Table S6). Among these, *WARS2* had the highest maximum SNP CADD score of 10.56, and the remaining 2 had a low risk of deleteriousness (CADD 6.85 for *RPS3AP12*, and CADD 3.06 for *RP11‐418J17.1*). The nearest genes for the chromosome 8 risk locus were *CDCA2* and *RP11‐219J21.1*, although these were distant, respectively 99 254 and 78 624 bases from the risk locus (Table S5).

#### eQTL Mapping

**Figure 2:**
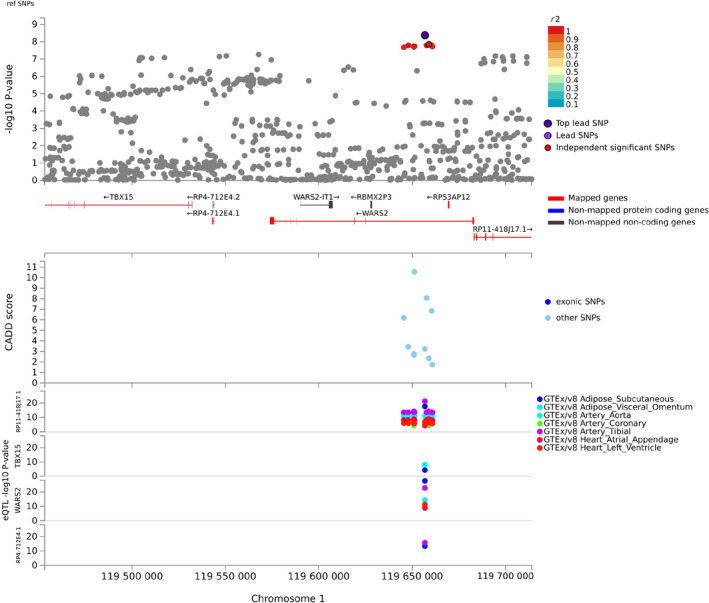
Regional plot of the chromosome 1 locus. Genes prioritized by FUMA are highlighted in red, and colors of genome‐wide significant SNPs are based on *r* ^2^. From the top: genome‐wide significance *P* value, CADD score, and eQTL *P* value. eQTLs are plotted for each gene, and colors are based on tissue types. CADD indicates combined annotation dependent depletion; eQTL, expression quantitative trait loci; GTE, Genotype‐Tissue Expression; RBMX2P3, RNA binding motif protein X‐Linked 2 pseudogene 3; SNP, single nucleotide polymorphism; TBX15, T‐box transcription factor 15; and WARS2, tryptophanyl tRNA synthetase 2, mitochondrial.

eQTL mapping consistently prioritized *WARS2* (protein coding, expressed in adipose, arterial, and cardiac tissues) and *RP11‐418J17.1* (antisense, expressed in adipose, arterial, and cardiac tissues), but additionally identified regulatory functions of the candidate variants on *TBX15* (protein coding, expressed in adipose tissues) and *RP4‐712E4.1* (lincRNA, expressed in adipose and arterial tissue) (Tables S6 and S7). No chromosome 8 genes were mapped using eQTLs. The locus plots, positional mapping, and corresponding eQTLs for chromosome 1 variants are summarized in Figure 2. Notably, the *TBX15* gene was also highlighted as the most strongly associated protein coding gene with adjusted PAT in MAGMA genome‐wide analysis (Figure S3).

#### Chromatin Interaction Mapping

**Figure 3:**
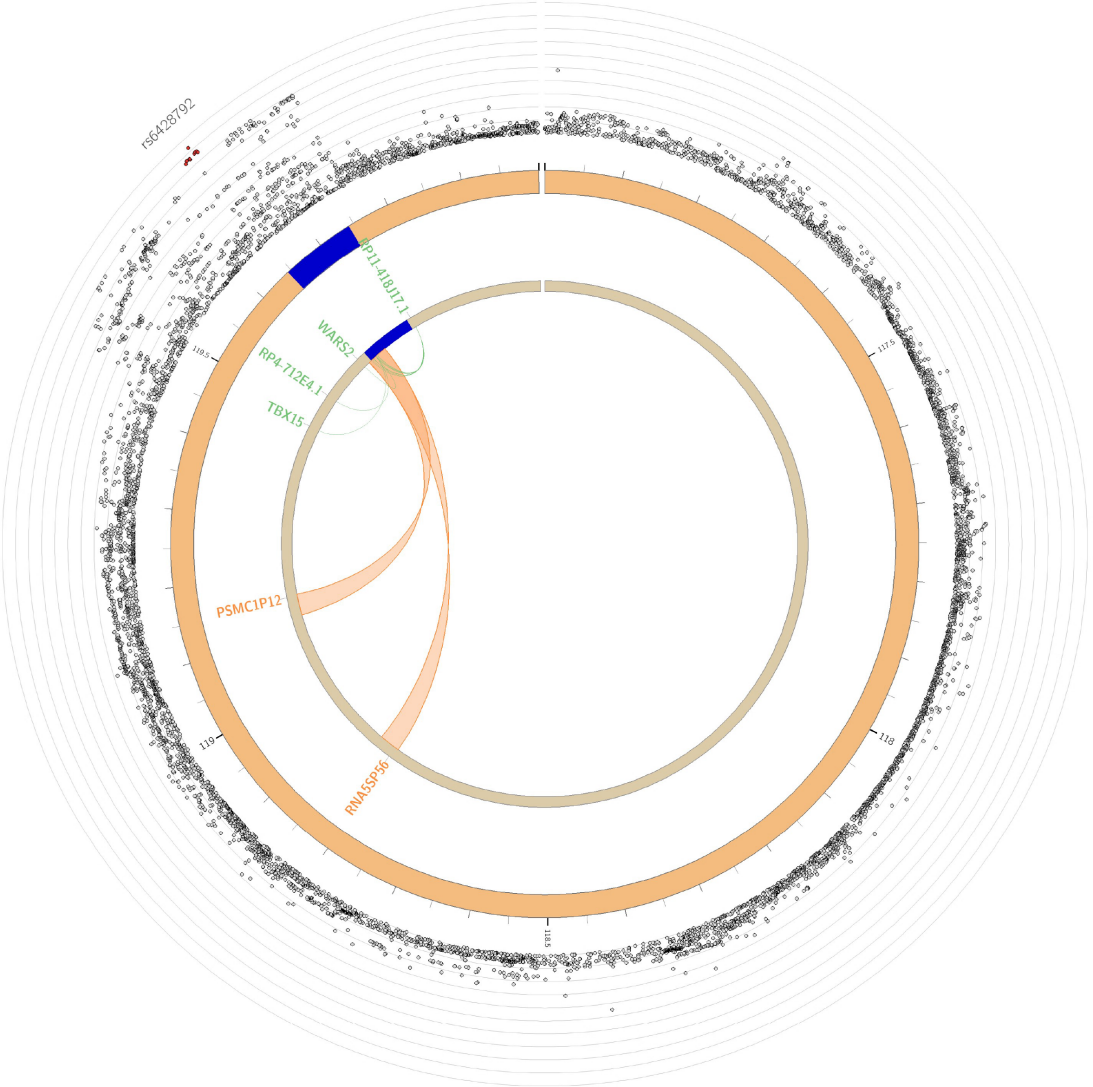
Chromatin interactions and expression quantitative trait loci of pericardial adipose tissue risk loci on chromosome 1. The outer layer displays genome‐wide association study *P* values, with the lead single nucleotide polymorphism labeled. Genes mapped by either expression quantitative trait loci or chromatin interactions are displayed in the innermost circle. Genes mapped by chromatin interactions are displayed in orange, expression quantitative trait loci in green, and those mapped by both in red. Orange links display chromatin interactions, and green links display expression quantitative trait loci. PSMC1P12 indicates proteasome 26S subunit, ATPase 1 seudogene 12; RNA5SP56, RNA, 5S ribosomal seudogene 56; TBX15, T‐box transcription factor 15; and WARS2, tryptophanyl tRNA synthetase 2, mitochondrial.

**Figure 4:**
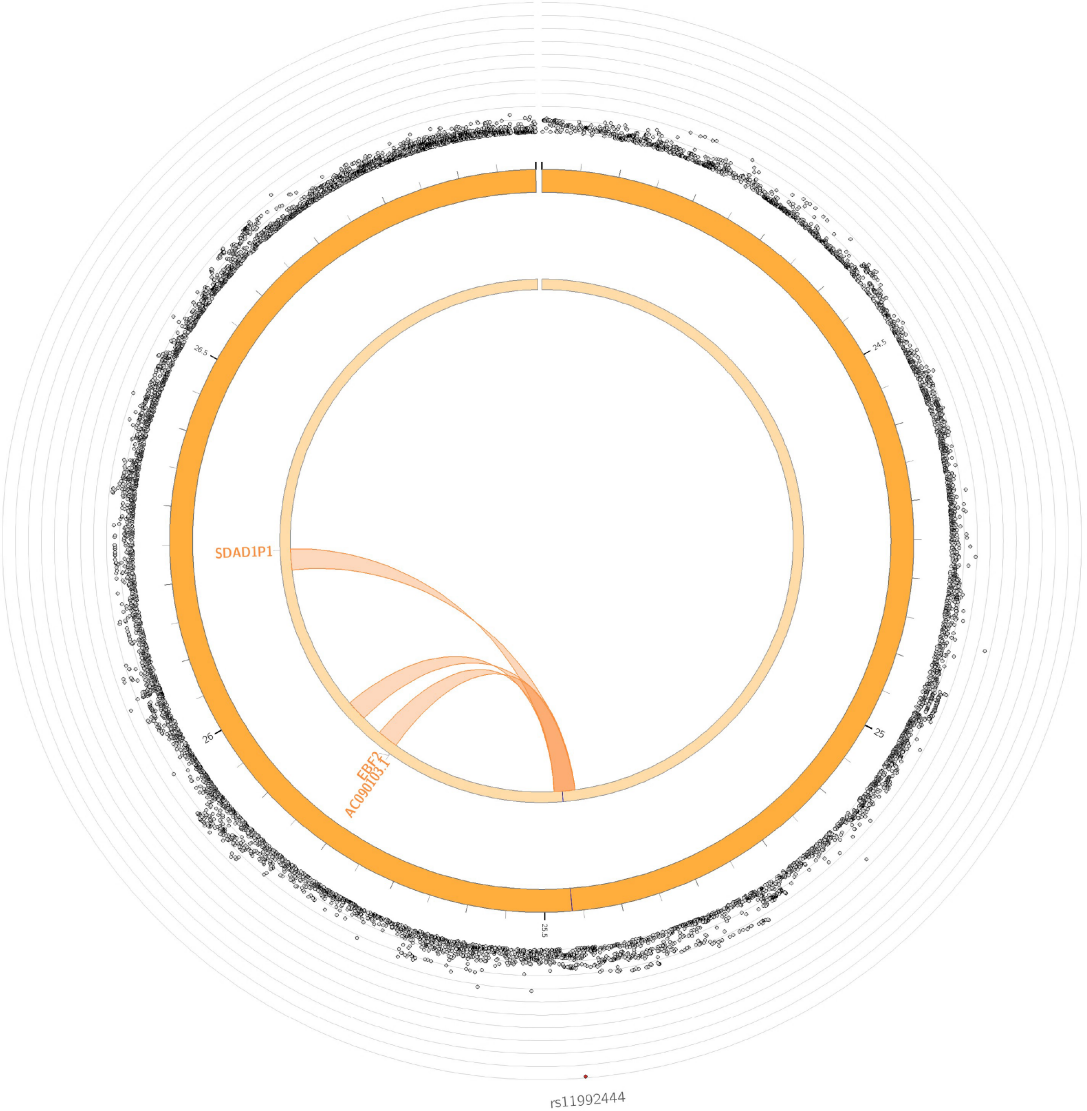
Chromatin interactions and expression quantitative trait loci of pericardial adipose tissue risk loci on chromosome 8. The outer layer displays genome‐wide association study *P* values, with the lead single nucleotide polymorphism labeled. Genes mapped by either expression quantitative trait loci or chromatin interactions are displayed in the innermost circle. Genes mapped by chromatin interactions are displayed in orange, expression quantitative trait loci in green, and those mapped by both in red. Orange links display chromatin interactions, and green links display expression quantitative trait loci. EBF2 indicates early B‐cell factor‐2; and SDAD1P1, SDA1 domain containing 1 pseudogene 1.

Finally, 11 chromatin interaction regions were identified (Table S8) mapping to 5 distinct genes (Table S6). These are depicted in Figure 3 and Figure 4. Using chromatin interaction mapping, a total of 3 genes were mapped in chromosome 8: *EBF2*, *AC090103.1*, and *SDAD1P1*. Among these, the protein coding *EBF2* gene appeared highly intolerant to loss of function (pLI 0.97).

#### Colocalization Analysis

Colocalization analysis was performed to explore whether risk variants for PAT were associated with gene expression in adipose, arterial, and cardiac tissues. Using cis‐eQTLs from GTEx version 8, associations were explored within 1 Mb of significant GWAS SNPs. In the discovery GWAS, evidence for colocalization was found in the *RP4‐712E4.1* locus in subcutaneous adipose tissue (PPH4=0.93) and tibial artery (PPH4=0.96; Table S9, Figures S4 and S5). For SNPs in the region surrounding RP4‐712E4.1, PAT risk and RP4‐712E4.1 tended to correlate, suggesting that increased PAT risk is associated with increased RP4‐712E4.1 expression (Figures S4D and S5D). These results were not duplicated in the replication data set. Sensitivity analysis confirmed that these colocalizations were robust to changes in the prior probability of a variant associating with both traits (ie, p12 prior; Figure S6). An additional locus of high PPH4 was found between the gene *CDCA2* in the LV, in both discovery and replication data sets, although these were driven by a single SNP (Figure S7). Multiple associations were found for loci where SNPs independently associated with PAT risk and gene expression in a region, including the *DOCK5* locus using the tibial artery eQTL (PPH3=0.93 in discovery and replication data sets) and in the *WARS2* and *RP11‐418J17.1* loci in all 7 tissues tested (PPH3≥0.99 throughout the discovery GWAS; Table S9).

#### Gene to Function

To understand putative biological mechanisms behind the potentially causal genes (*TBX15*, *WARS2*, *EBF2*), gene‐to‐function mapping was performed in FUMA. A visual representation of normalized gene expression across tissue types is depicted in Figure S8, highlighting the elevated expression of *EBF2* and *TBX15* in adipose tissue, with only *EBF2* specifically expressed in visceral omental adipose tissue. Differentially expressed gene analyses did not identify any statistically significant differences in gene expression across tissue types (Table S10). The gene‐set enrichment and pathway analyses did not yield any significant results.

A phenome‐wide association study was performed for protein coding potentially causal genes. The 2 prioritized genes on chromosome 1, *TBX15* and *WARS2*, were associated with similar phenotypes, including male pattern baldness, white blood cells, measures of overall adiposity and its distribution, bone mineral density, and height (Figure S9, Table S11). The prioritized chromosome 8 gene, *EBF2*, was associated with traits relating to adiposity and its distribution and height but was also associated with blood pressure traits. An association was also noted with inguinal hernias. The results are presented in Figure S10 and Table S11. In mice, homozygous loss of function in both *EBF2* and *WARS2* have been associated with embryonic lethality, whereas heterozygous loss‐of‐function mutations in *EBF2* have been associated with a variety of cardiac, spleen, vascular, and other malformations. The full list of mouse phenotypes is reported in Table S12.

### Heritability and Phenotypic Associations

#### Heritability and Genetic Correlations

The genome‐wide heritability (h^2^_g_ SNP) of adiposity‐adjusted PAT was estimated at 9.15% (standard error, 2.49%). The genetic correlations of adjusted PAT are displayed in Table S13. There was no significant correlation with adiposity measures, which is expected given the adjustment for these measures in the GWAS analysis. A nominally significant correlation was noted between adjusted PAT and heart failure (genetic correlation [rG]=0.36, standard error [se]=0.18, *P*=0.048). No further correlations were discovered with other cardiovascular outcomes, and no associations were significant after accounting for multiple testing.

#### Mendelian Randomization

The instrumental variants extracted for MR analyses corresponded with the 2 prioritized lead variants at the 2 risk loci. *F* statistics were 34.5 for rs6428792 and 50.3 for rs11992444, indicating adequate instrument strength.

Higher genetically predicted adjusted PAT was associated with lower LVEDV (β, −1.04 [95% CI, −1.88 to −0.19]; *P*=0.016) and LV end‐systolic volume (β, −0.91 [95% CI, −1.74 to −0.08]; *P*=0.032). There was no significant association between genetically predicted PAT and LV stroke volume (β, −0.72 [95% CI, −1.73 to 0.07]; *P*=0.072), LV ejection fraction (β, 0.23 [95% CI, −0.64 to 1.11]; *P*=0.602), and LV mass:LVEDV ratio (β, 1.14 [95% CI, −0.28 to 2.55]; *P*=0.115).

**Figure 5:**
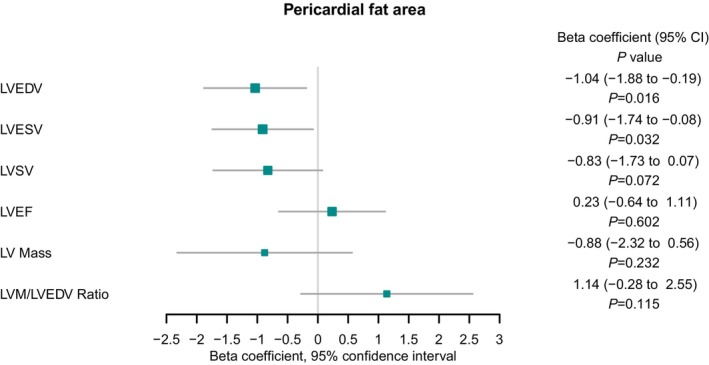
Inverse‐variance weighted Mendelian randomization analysis exploring the association between pericardial fat area (pericardial adipose tissue) and left ventricular (LV) end‐diastolic volume (LVEDV), LV end‐systolic volume (LVESV), LV ejection fraction (LVEF), LV mass (LVM), and LV mass to end‐diastolic volume ratio (LVM:LVEDV). LVSV indicates left ventricular stroke volume.

**Figure 6:**
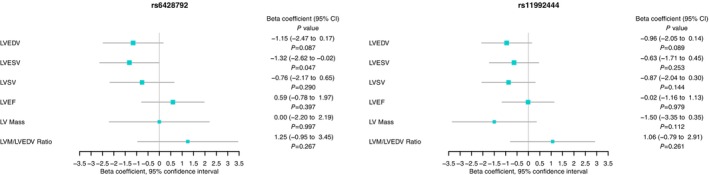
Single–single nucleotide polymorphism Mendelian randomization analysis (Wald ratio method) exploring the association between pericardial fat area (pericardial adipose tissue) through rs6428792 and rs11992444 and left ventricular (LV) end‐diastolic volume (LVEDV), LV end‐systolic volume (LVESV), LV ejection fraction (LVEF), LV mass (LVM), and LV mass to end‐diastolic volume ratio (LVM:LVEDV). LVSV indicates left ventricular stroke volume.

The results of the MR analyses are summarized in Figure 5 and Table S14. Single‐SNP analysis revealed consistency in effect estimate directions with the main analysis and between both instrumental variants, as depicted in Figure 6.

#### Sensitivity Analyses

The meta‐analysis GWAS resulted in 185 SNPs that passed the GWAS *P* value threshold (5×10^−8^) mostly in chromosome 1 and 2 and 1 in chromosome 8 (Table 3). The leading SNPs are rs6428792 (chromosome 1), rs1430788 (chromosome 2), and rs1199244 (chromosome 8) which match the GWAS discovery and replication summaries. rs1430788 (chromosome 2) was neither significant in the discovery nor in the replication GWAS, although it was among the leading SNPs in the meta‐analysis.

**Table 3:** Meta‐analysis Genome‐wide Association Studies Summary Statistics for the Lead SNPs Using the Metal Tool

SNP	Chromosome	BP	Allele 1	Allele 2	Effect	Standard error	*P* value	Direction
rs6428792	1	119 656 867	A	G	−0.0092	0.0014	1.67E‐11	…
rs143078898	2	229 994 086	T	C	−0.0133	0.0023	1.53E‐08	…
rs11992444	8	25 464 690	T	G	−0.0127	0.0013	8.77E‐22	…

BP indicates base pair; and SNP, single nucleotide polymorphism.

The results of the more relaxed GWAS (without adjustment for fat measures) are presented in Table S15. rs11992444 (chromosome 8) SNP that was replicated in the adjusted model and in the meta‐analysis was also significant in the relaxed GWAS. In addition, the rs143078898 (chromosome 2) SNP that was significant in the meta‐analysis GWAS was also significant in the relaxed GWAS analysis.

## Discussion

This study is the largest individual‐level GWAS to date exploring the polygenic basis and genetic architecture of PAT. To add to previous literature, we specifically aimed to disentangle PAT from multiple other biometric measures of total adiposity and its distribution to isolate specific determinants of preferential fat deposition in the pericardial compartment. This strategy yielded a total of 11 genome‐wide significant variants, with 2 lead uncorrelated SNPs relating to 2 genomic risk loci. These were mapped to 10 potentially causal genes using positional, eQTL, and chromatin interaction mapping. Among these, 3 protein coding genes were identified: *TBX15*, *WARS2*, and *EBF2*. For the latter 2 genes, enrichment analyses determined significant tissue‐specific eQTLs and chromatin interactions in both adipose and cardiac tissue, supporting an overlapping physiology in these tissue types. Importantly, we also found that the proportion of phenotypic variance explained by the genotype was 9.1%, indicating a relatively high genetic determination of proportionally greater PAT deposition.

To date, only 2 genome‐wide association studies^18^, ^19^ have been performed exploring the polygenic basis of PAT. Fox et al^18^ explored the genetic determinants of PAT adjusted for visceral fat volume, WHR, and BMI in 5487 participants of the Framingham Heart Study, uncovering 1 single genome‐wide significant variant at 1 locus (rs10198628 mapped to the *TRIB2* gene). In our relaxed GWAS, this SNP was only nominally associated with PAT (*P* value=0.029). The result was similar in the main GWAS analysis adjusted for fat measures (*P* value=0.037) and in the meta‐analysis (*P* value=0.012). Chu et al^19^ explored the genetic determinants of PAT, adjusted for height and weight only, in a cohort of 18 332 participants that included individuals in the study by Fox et al. A total of 3 genome‐wide significant variants were identified (rs6587515 mapped to the *ENSA* gene, rs1650505 mapped to the *EBF1* gene, and rs10198628 mapped to the *TRIB2* gene). Among them, 1 was replicated from the study by Fox et al (rs10198628 [chromosome 2]). In our “relaxed” GWAS, rs6689335 (*P*=0.320), rs6587515 (*P*=0.220), and rs10198628 (*P*=0.015) were replicated. In the main GWAS analysis with adjustment for fat measures, rs6689335 was not associated with PAT (*P*=0.900) and neither was rs6587515 (*P*=0.150), whereas rs10198628 was (*P*=0.160). In the meta‐analysis, rs6689335 (*P*=0.657) and rs6587515 (*P*=0.383) were not associated with PAT, whereas rs10198628 (*P*=0.011) was nominally significant but did not pass GWAS threshold. This discrepancy is likely to relate to the lack of sample overlap and more comprehensive adjustment for measures of total and relative adipose tissue distribution in our analysis. Importantly, we carried out a replication analysis in an independent subset of UK Biobank participants who were excluded from the discovery analysis. This replicated all the genome‐wide significant signals at Bonferroni‐adjusted *P* value, increasing confidence in the validity of our results.

Among the genome‐wide significant variants discovered, 10 of the 11 were located in a single genomic risk locus on chromosome 1. Among these, 1 single lead variant was retained (rs6428792). Positional mapping identified 3 potential causal genes, eQTL mapping identified 4 potential causal genes (2 overlapping) and chromatin interaction using Hi‐C data from the LV identified 2 further potential causal genes. Colocalization analysis suggested that, for all the genes in the implicated region in chromosome 1, the risk of PAT in both subcutaneous adipose and tibial arterial regions were associated with increase gene expression of RP4‐712E4.1, a long noncoding RNA, at this locus. For the chromosome 8 variant (rs11992444), positional and eQTL mapping did not identify any genes, and the colocalization analysis was inconclusive. However, chromatin interaction mapping using Hi‐C data from the LV identified 3 potentially causal genes. Overall, among the identified potentially causal genes at both loci, 5 had been previously associated with BMI‐adjusted adiposity distribution traits (*TBX15*, *WARS2*, *EBF2*, *PSMC1P12*, *RNA5SP56*), and 1 gene, *SDAD1P1*, has been previously associated with red cell distribution width. The remaining 4 genes had no previously reported associations.

The potentially causal protein coding genes have been implicated in a variety of physiological pathways. *EBF2* is known to play a key role in activating the expression of brown fat–selective genes in adipocytes.^55^ *WARS2* encodes a cytoplasmic form of tryptophanyl‐tRNA synthetase, which has been shown to play a central role in angiogenesis, including cardiac angiogenesis.^56^ In mouse models, a reduction of *WARS2* gene function was shown to lead to reduced food intake and depot‐specific changes in fat mass and brown fat distribution.^57^ Similarly, *TBX15* activation has been implicated in the preferential distribution of abdominal adiposity^58^ as well as in adrenergic‐induced adipocyte browning.^59^ Generally, white adipose tissue is considered predominantly an inactive energy storage, whereas brown adipose tissue contains a higher concentration of mitochondria and expresses UCP1 (uncoupling protein 1), a protein that enables its metabolic use and thermogenesis.^60^ PAT is considered predominantly a white adipose tissue depot, although it is known to have higher expression of UCP1 compared with white adipose tissue in the rest of the body. The results of our study and functional annotation suggest that a reduced propensity toward fat browning likely contributes to higher proportional PAT deposition. Indeed, both lead variants in this study were inversely associated with PAT, and unaligned eQTL mapping displayed a predominantly inhibitory role of the unaligned variants on *WARS2*, but an enhancing role on *TBX15*. Thus, aligning the variants toward greater PAT would suggest an enhancing role on *WARS2*, and an inhibitory action on *TBX15*, both of which are consistent with a phenotype of inhibited adipose tissue browning. This is mechanistically consistent with previous observational work outlining an inverse association between brown adipose tissue and visceral adiposity deposition.^61^

To relate the genetic data with potential biological consequences of PAT, we examined genetic correlation analyses and performed MR. A genetic correlation was observed between adjusted PAT and heart failure, consistent with previous evidence linking PAT with heart failure^6^ and adverse cardiac structure and function independent of overall adiposity.^9^ Building on these observational data, we performed MR analyses to elucidate the potential causal relevance of PAT on cardiac structure and function. This revealed an association of higher PAT with lower LVEDV, lower LV end‐systolic volume, and a suggestive result for lower LV stroke volume. This is broadly reflective of a reduction in ventricular chamber volume, consistent with remodeling patterns seen in aging^62^ and in heart failure with a preserved ejection fraction.^63^ Beyond the reduction in LV volumes and stroke volume, the aging heart with a preserved ejection fraction phenotype is characterized by lower LV mass attributed to cardiomyocyte attrition,^63^, ^64^ typically occurring to a lesser proportion to the reduction in volumes, leading to an increased LV mass:LVEDV ratio reflecting greater concentricity.^63^ In this phenotype, LV ejection fraction would be expected to remain similar or paradoxically increase with the rise in concentricity.^65^ Although not all of these associations were statistically significant, the directionality of the MR results is consistent with remodeling in a heart failure with a preserved ejection fraction cardiac phenotype. This is consistent with the cardiac remodeling pattern that has previously been associated with PAT in observational studies.^6^, ^66^, ^67^

We acknowledge some limitations. Despite being the largest currently available GWAS of PAT, the number of loci discovered remains small. In addition, because of the restricted sample size, the analysis was restricted to variants with a minor allele frequency>1%. Incorporation of rare variants in further analyses when larger sample sizes are available might enhance genetic discovery. Finally, the UK Biobank population was restricted to European ancestry; therefore, further research is warranted in populations of other ancestries.

In summary, the results of this study enhance the current knowledge about the genetic basis of preferential PAT deposition, prioritize a number of potentially causal genes that might exert influence through the modulation of adipose tissue browning, and provide genetic evidence to support causal relevance of PAT on cardiac structure and function that might contribute to heart failure risk.

## Sources of Funding

Salih is supported by a British Heart Foundation project grant (PG/21/10619). Ardissino is funded by a National Institute for Health and Care Research for an Academic Clinical Fellowship. Barts Charity (G‐002346) contributed to fees required to access UK Biobank data (access application 2964). Petersen acknowledges the British Heart Foundation for funding the manual analysis to create a cardiovascular magnetic resonance imaging reference standard for the UK Biobank imaging resource in 5000 cardiovascular magnetic resonance scans (http://www.bhf.org.uk; PG/14/89/31194), support from the National Institute for Health and Care Research Biomedical Research Centre at Barts, and support from the “SmartHeart” Engineering and Physical Sciences Research Council (EPSRC) program grant (http://www.nihr.ac.uk; EP/P001009/1). Raisi‐Estabragh was supported by a British Heart Foundation Clinical Research Training Fellowship (FS/17/81/33318). Petersen and Szabo have received funding from the European Union's Horizon 2020 research and innovation program under grant agreement No. 825903 (an EU‐Canada joint infrastructure for next‐generation multi‐Study Heart Research [euCanSHare] project). This article is supported by the London Medical Imaging and Artificial Intelligence Centre for Value Based Healthcare, which is funded by the Data to Early Diagnosis and Precision Medicine strand of the government's Industrial Strategy Challenge Fund, managed and delivered by Innovate UK on behalf of UK Research and Innovation. The views expressed are those of the authors and not necessarily those of the Artificial Intelligence Centre for Value Based Healthcare Consortium members, the National Health Service, Innovate UK, or the UK Research and Innovation. This work was supported by Health Data Research UK, an initiative funded by UK Research and Innovation, Department of Health and Social Care (England) and the devolved administrations, and leading medical research charities. The funders provided support in the form of salaries for the authors as detailed previously but did not have any additional role in the study design, data collection and analysis, decision to publish, or preparation of the article.

## Disclosures

Professor Petersen provides consultancy to Circle Cardiovascular Imaging Inc., Calgary, Alberta, Canada. The remaining authors have no disclosures to report.

## Supporting information

Tables S1–S15

Figures S1–S10
