# Supplementary material for: Genome‐Wide Association Study of Pericardial Fat Area in 28 161 UK Biobank Participants
Source: J Am Heart Assoc. 2023 Oct 27;12(21):e030661. doi: 10.1161/JAHA.123.030661 (PMC10727393; doi:10.1161/JAHA.123.030661)

Supplemental Material

Supplemental Table Legends:

Please see the separate Excel file for the supplemental tables.

Table S1 – Candidate SNPs, defined as all genome-wide significant SNPs associated with adjusted PAT ($p<5x10^{-8}$) and additional highly correlated SNPs identified via 1000G Phase 3 data.

Table S2 – Genomic risk loci of interest, respective lead SNPs and independent significant SNPs in the locus.

Table S3 – Lead SNPs identified from genome-wide SNPs at $r^2<0.1$. Genomic locus: the index of genomic risk loci specified in Supp Tab 3. #Ind. Sig. SNPs: Independent significant SNPs which are in LD with the corresponding lead SNPs at $r^2<0.1$

Table S4 – Phenotypic associations for lead SNPs and additional closely correlated SNPs ($r^2>0.8$) available in GWASCatalog

Table S5 – Variant annotation for all candidate SNPs using ANNOVAR.

Table S6 – Genes prioritized using positional mapping, eQTL mapping (immune cells, arterial, adipose and cardiac tissue types) and chromatin interaction (aorta, right ventricle, left ventricle) mapping.

Table S7 –Tissue-specific eQTLs discovered in adipose, heart and arterial tissue for genomic risk loci (FDR<0.05).

Table S8 – Significant chromatin interactions (Hi-C) within aorta, left ventricle and right ventricle discovered for genomic risk loci (FDR<0.05).

Table S9 – Results for colocalisation analysis. Table shows results for all genes within 1Mb of a significant GWAS hit, tested with expression quatitative trait loci from GTEx8. PPH4 > 0.8 suggests colocalisation of GWAS risk and gene expression. Abbreviations: nsnps – number of snps tested at a locus; PP.H0-4.abf – posterior probability of hypothesis 0-4 respectively; sum_PPH3_PPH4 - sum of posterior hypotheses 3 and 4; ratio_PPH4_PPH3 – ratio of posterior hypothesis 4 to posterior hypothesis 3.

Table S10 – Differential gene expression analysis (DEG) comparing expression of candidate genes in each tissue type, versus all other tissue types.

Table S11– Phenome-wide associations for EBF2, TBX15 and WARS2 gene among currently available studies on GWASAtlas.

Table S12 – List of prior associations for loss-of-function in potential causal genes with phenotypes in mouse studies, sources using International Mouse Phenotyping Consortium (IMPC) data.

Table S13 – Genetic correlations between PAT and adiposity traits (trunk fat mass ad percentage, whole body fat mass), cardiovascular risk factors (hypertension, diabetes, obesity), and cardiovascular outcomes (coronary heart disease, coronary event, heart failure, stroke, atrial fibrillation and flutter, and cardiac death).

Table S14 – Mendelian randomization analysis exploring the association between genetically-predicted pericardial fat area (PAT), overall and in single-SNP analysis, and left ventricular end diastolic volume (LVEDV), end systolic volume (LVESV), stroke volume (LVSV), ejection fraction (LVEF), mass (LVM) and mass to end diastolic volume ratio (LVM/LVEDV Ratio).

Table S15 – Genome-wide significant variants without adjustment for fat measures. The table displays beta coefficients with standard errors, and p-value estimates. Allele 1 is the effect allele.

Figure S1 – Genome-wide significant variants for pericardial fat area after adjusting for sex, age, age^2, age*sex, 10 genetic principal components (PCs), assessment centre, genotype array, and 2 PCs reflecting BMI, WHR, whole body fat mass, trunk fat mass, body fat percentage. The dashed line represents the genome-wide significance threshold, $p<5x10^{-8}$.

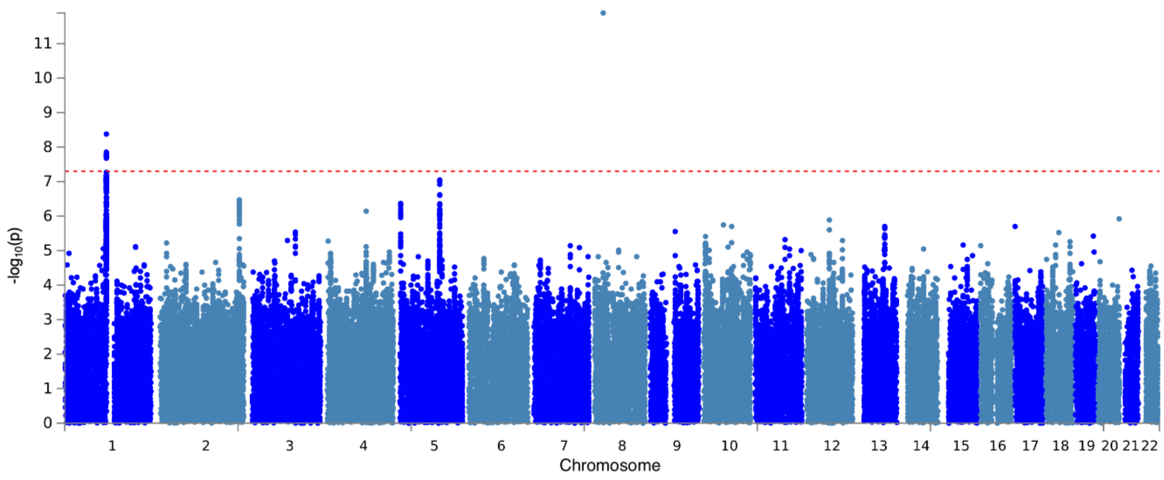

Figure S2 – Q-Q plot of for association of genetic variants with pericardial fat area after adjusting for sex, age, age^2, age*sex, 10 genetic principal components (PCs), assessment centre, genotype array, and 2 PCs reflecting BMI, WHR, whole body fat mass, trunk fat mass, body fat percentage. The dashed line represents the null hypothesis.

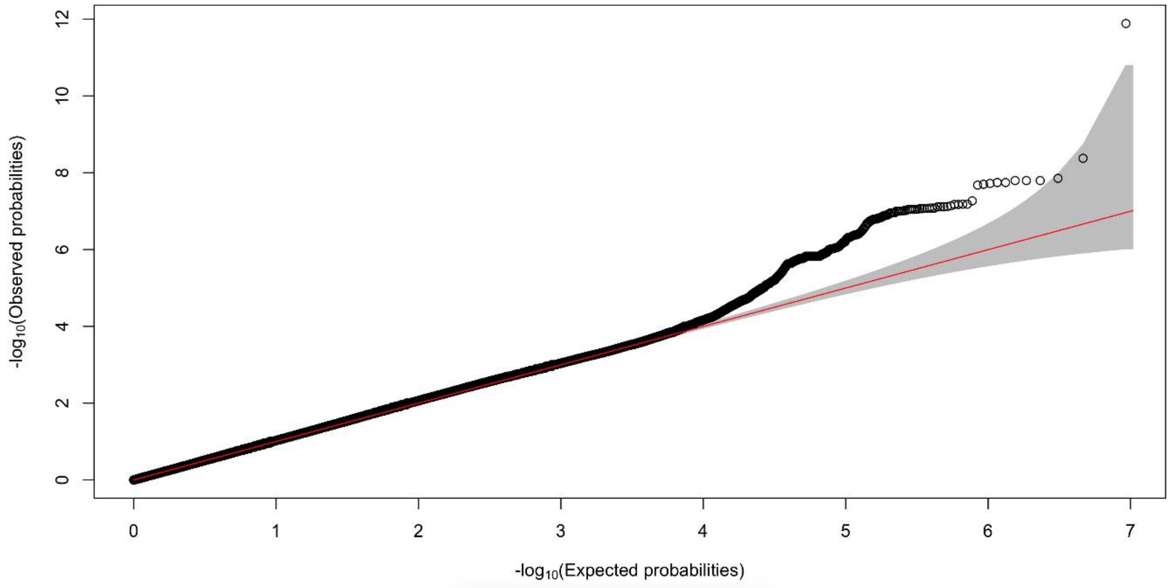

Figure S3 – Manhattan plot of the MAGMA gene-based test. The red line represents genome wide significance. With the inclusion of 19,086 protein coding genes, this was defined at P = 0.05/19086 = 2.62×10^{-6}

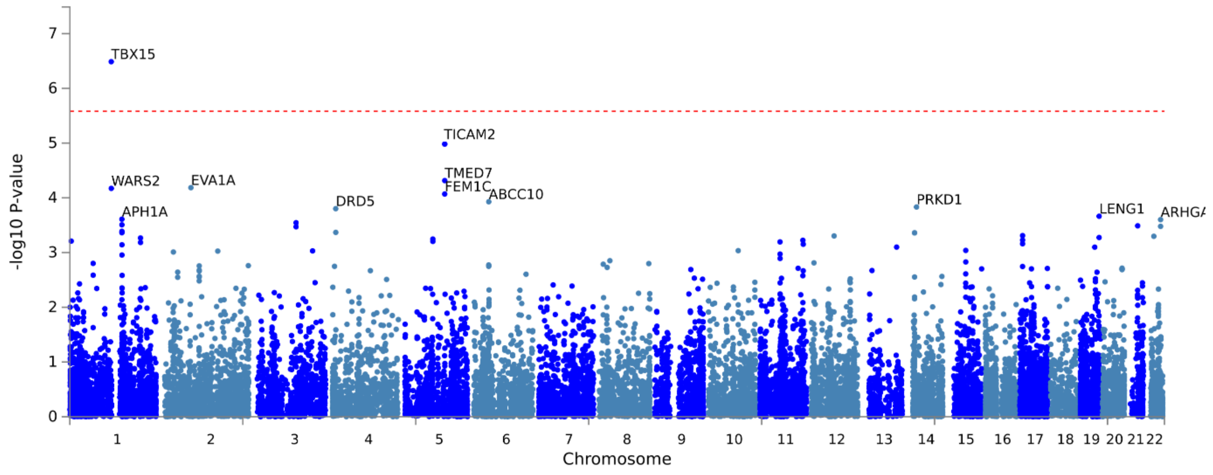

Figure S4 – Results from colocalisation analysis of RP4-712E4.1 in subcetaneous adipose tissue. A and C show regional association plots for Regional association plots for GWAS and eQTL respectively, with chromosome position as mapped in GRCh38. Comparison of betas (B), and p-values (D) from eQTLs and GWAS are shown, with overlay of Pearson's correlation)

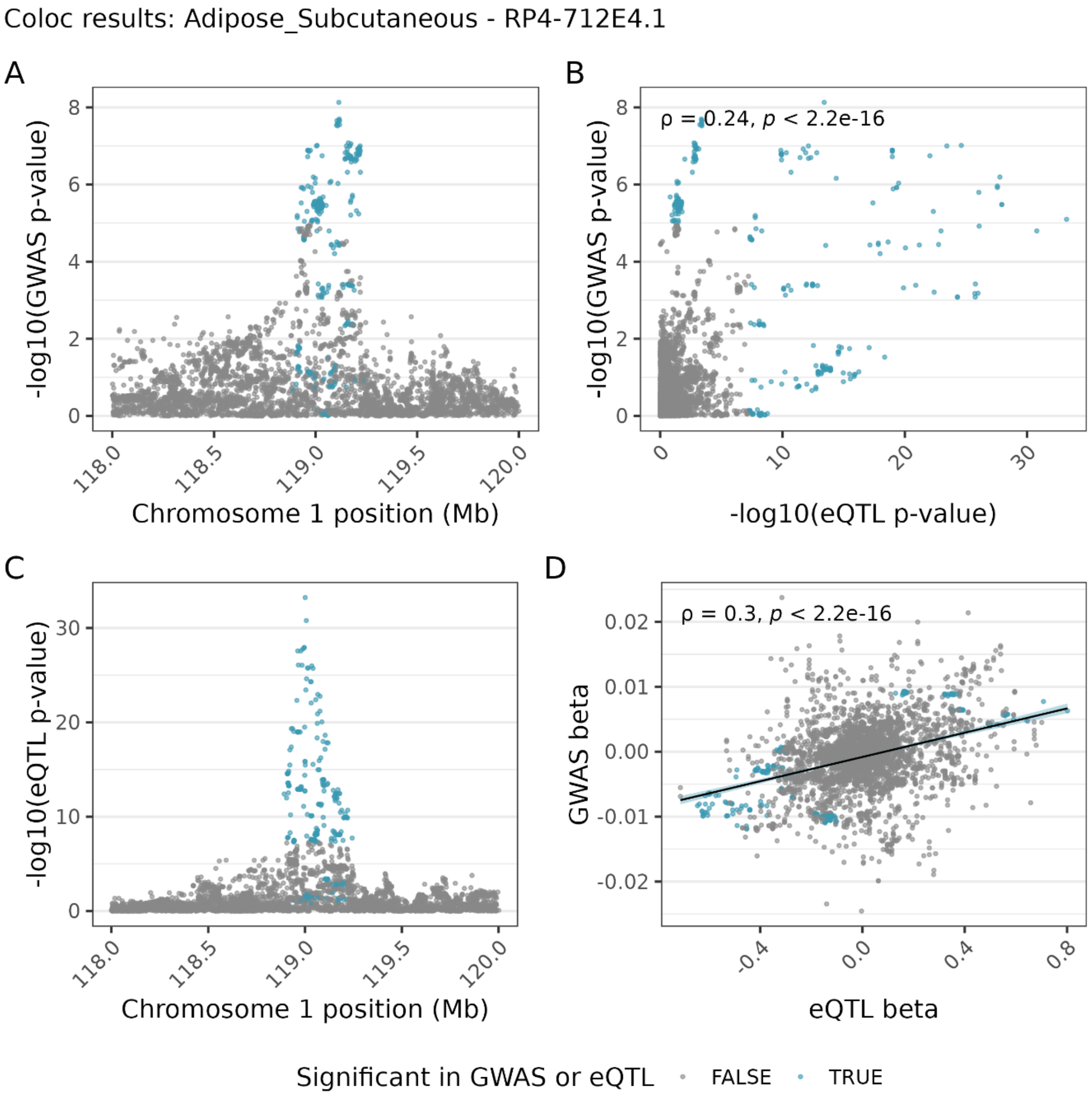

Figure S5 – Results from colocalisation analysis of RP4-712E4.1 in tibial artery. A and C show regional association plots for regional association plots for GWAS and eQTL respectively, with chromosome position as mapped in GRCh38. Comparison of betas (B), and p-values (D) from eQTLs and GWAS are shown, with overlay of Pearson's correlation).

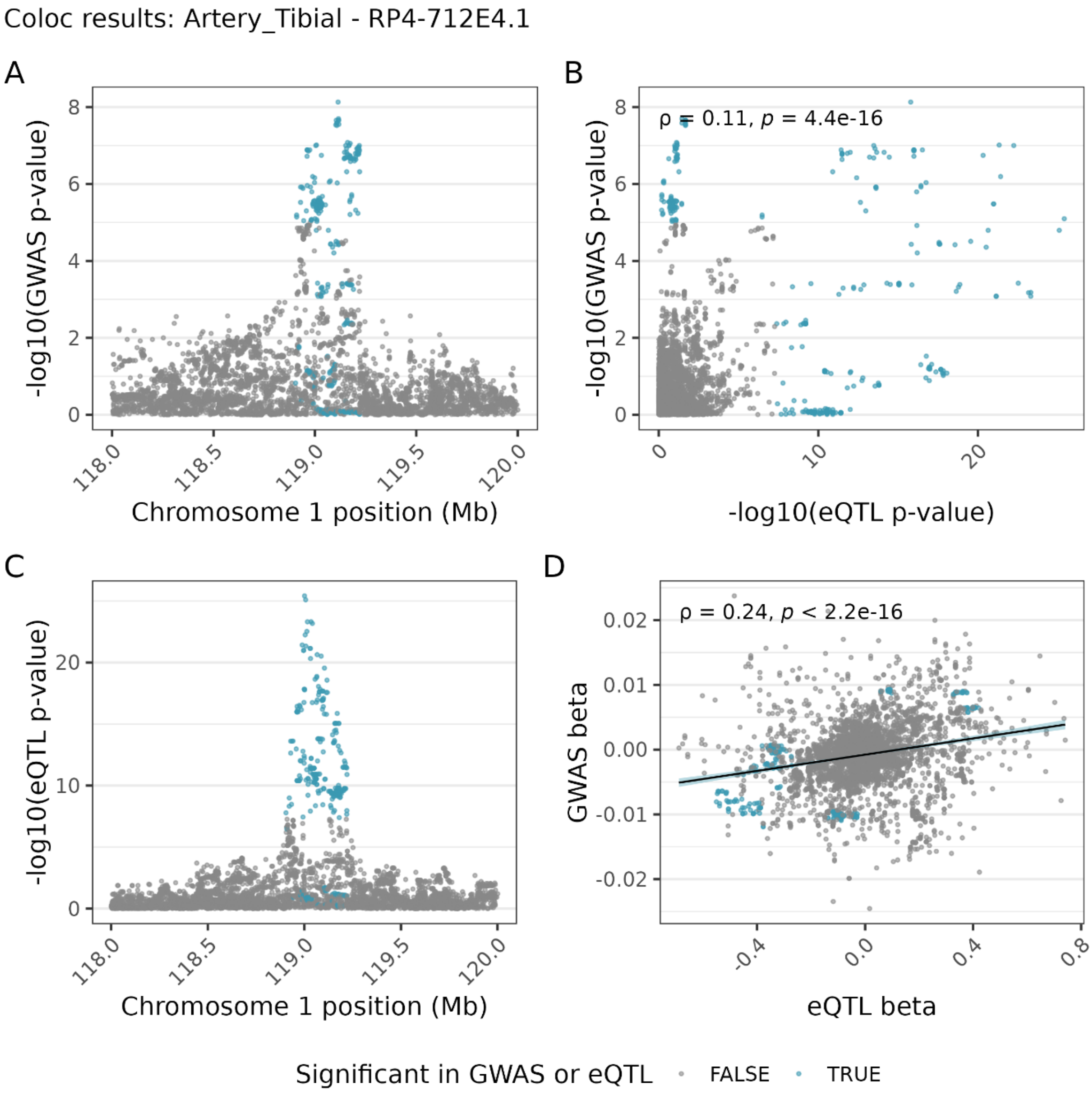

Figure S6. Results of sensitivity analysis showing prior and posterior probability distributions as a function of the p_{12} prior for: A – RP4-712E4.1 in subcutaneous adipose tissue; B – RP4-712E4.1 in tibial artery; and C – CDCA2 in the left ventricle.

A

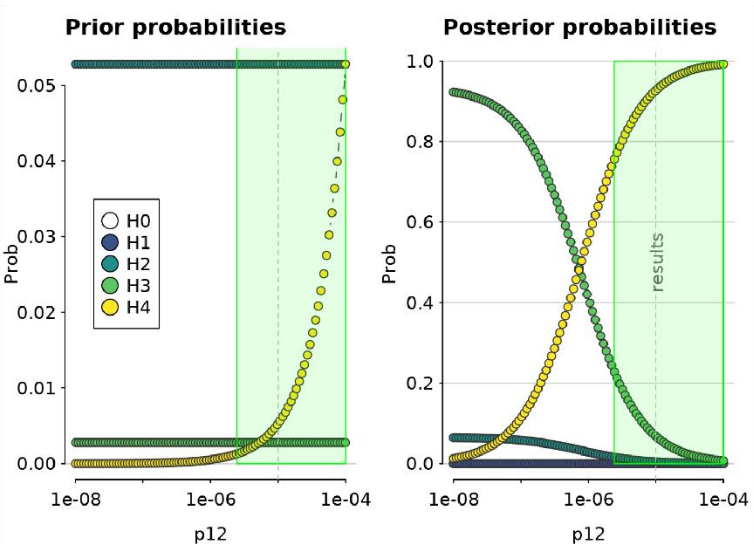

B

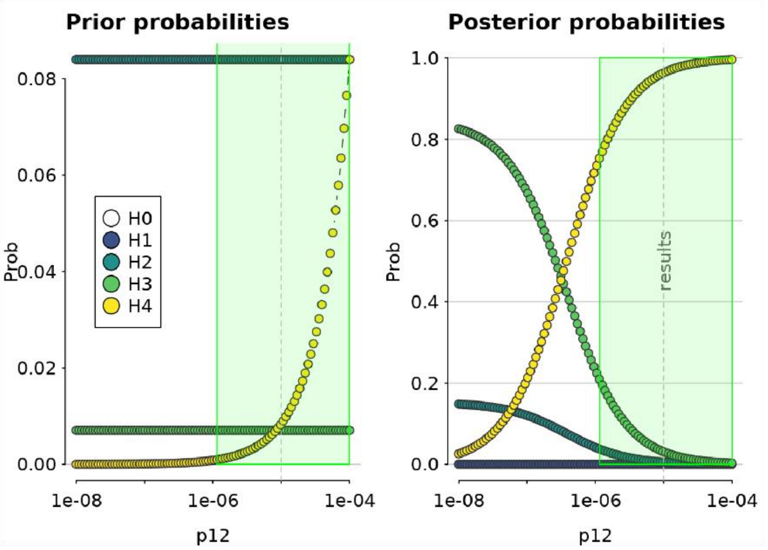

C

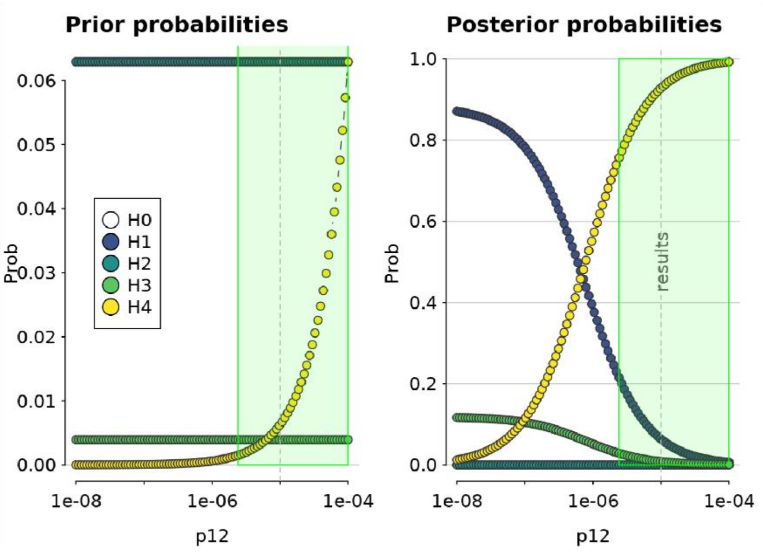

Figure S7. Results from colocalisation analysis of CDCA2 in the left ventricle. A and C show regional association plots for regional association plots for GWAS and eQTL respectively, with chromosome position as mapped in GRCh38. Comparison of betas (B), and p-values (D) from eQTLs and GWAS are shown, with overlay of Pearson's correlation). Results are driven by a single SNP and are therefore less likely to be a true colocalisation.

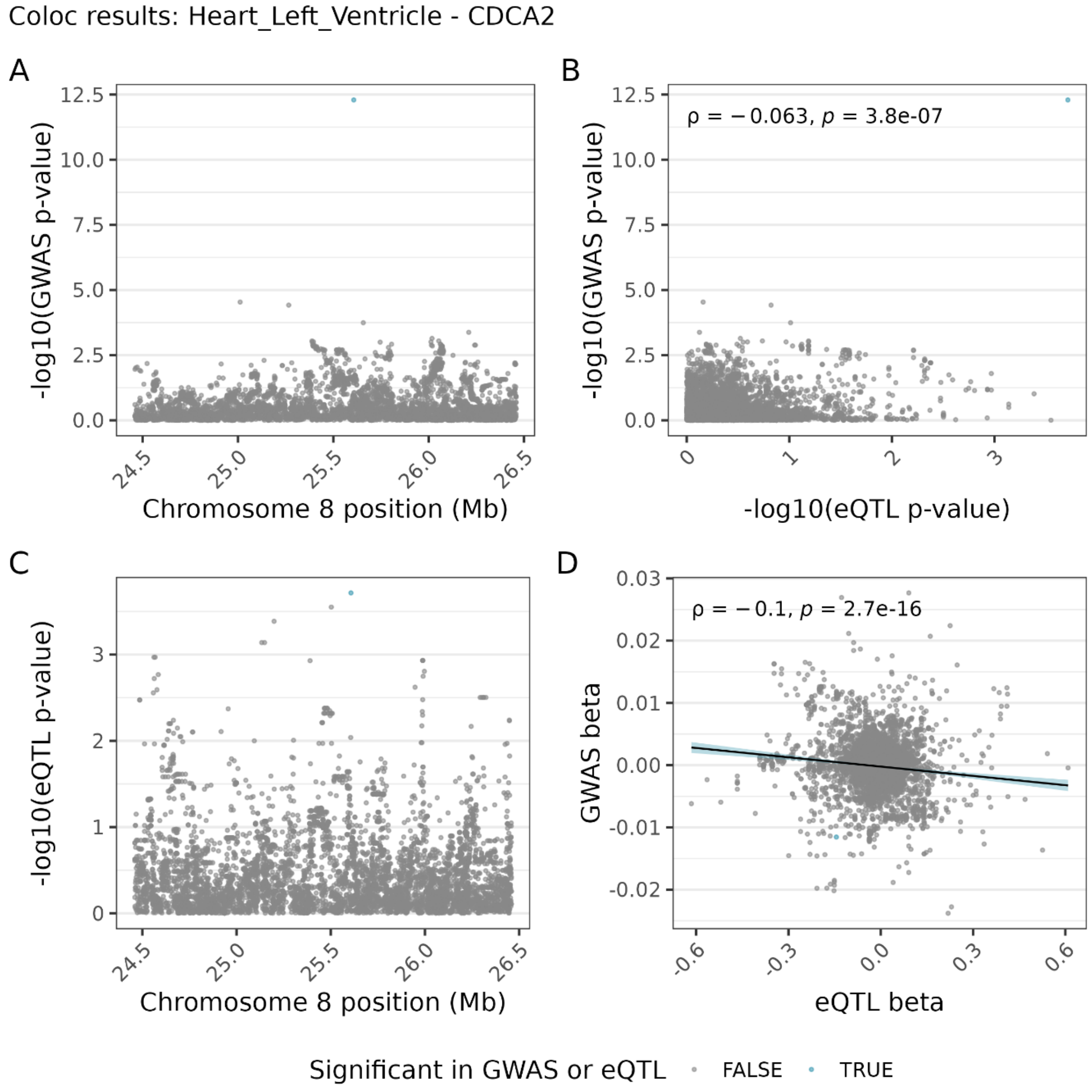

Figure S8 – Average normalised expression of all mapped genes in 54 tissue types extracted from GTEx v8. Red indicates higher gene expression, normalised per gene.

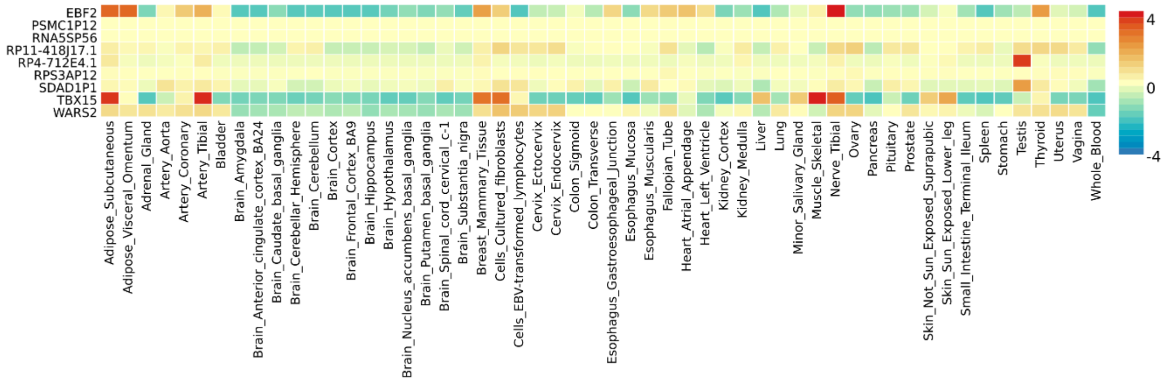

Figure S9 – Phenome-wide associations for TBX15 and WARS2 gene among currently available studies on GWASAtlas. Coloring corresponds to phenotype cluster, summarized in labels on the right. Only associations with a minimum p-value of 0.05 are displayed.

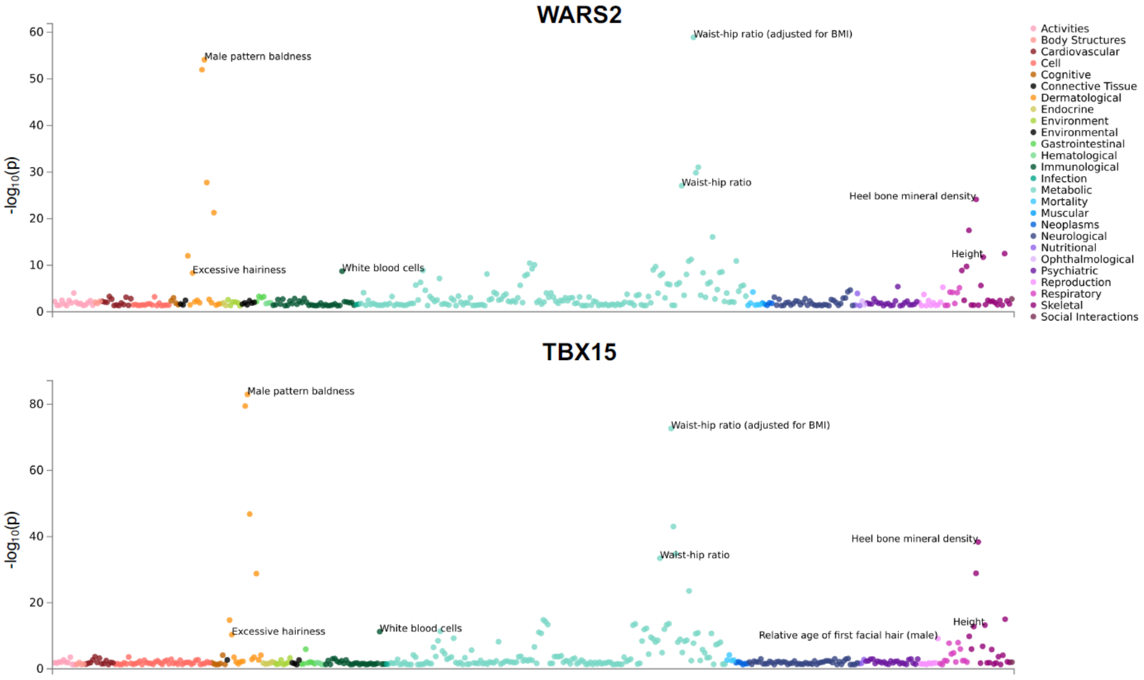

Figure S10 – Phenome-wide associations for EBF2 gene among currently available studies on GWASAtlas. Coloring corresponds to phenotype cluster, summarized in labels on the right. Only associations with a minimum p-value of 0.05 are displayed.

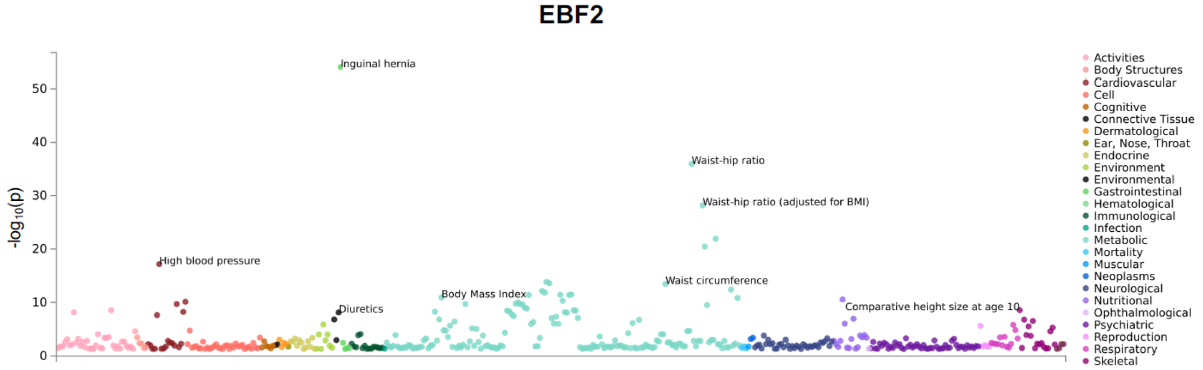

Supplement: Supplementary file 2 — Figures S1–S10 [file JAH3-12-e030661-s001.pdf]
